# Adverse events in spine surgery in Sweden: A comparison of patient claims data and national quality register (Swespine) data

**DOI:** 10.3109/17453674.2011.636673

**Published:** 2011-11-25

**Authors:** Annica Öhrn, Anders Olai, Hans Rutberg, Per Nilsen, Hans Tropp

**Affiliations:** ^1^Department of Medical and Health Sciences, Linköping University, Linköping; ^2^Department of Spine Surgery, Faculty of Health Sciences, Linköping University, Linköping; ^3^Patient Safety Unit, County Council of Östergötland, Sweden

## Abstract

**Background and purpose:**

Our knowledge of complications and adverse events in spinal surgery is limited, especially concerning incidence and consequences. We therefore investigated adverse events in spine surgery in Sweden by comparing patient claims data from the County Councils' Mutual Insurance Company register with data from the National Swedish Spine Register (Swespine).

**Methods:**

We analyzed patient claims (n = 182) to the insurance company after spine surgery performed between 2003 and 2005. The medical records of the patients filing these claims were reviewed and compared with Swespine data for the same period.

**Results:**

Two-thirds (119/182, 65%) of patients who claimed economic compensation from the insurance company were registered in Swespine. Of the 210 complications associated with these 182 claims, only 74 were listed in Swespine. The most common causes of compensated injuries (n = 139) were dural lesions (n = 40) and wound infections (n = 30). Clinical outcome based on global assessment, leg pain, disability, and quality of health was worse for patients who claimed economic compensation than for the total group of Swespine patients.

**Interpretation:**

We found considerable under-reporting of complications in Swespine. Dural lesions and infections were not well recorded, although they were important reasons for problems and contributed to high levels of disability. By analyzing data from more than one source, we obtained a better understanding of the patterns of adverse events and outcomes after spine surgery.

A Swedish study estimated that surgical disciplines accounted for approximately 62% of preventable adverse events in Swedish hospitals (Soop et al. 2009). The overall prevalence of preventable adverse events resulting in patient injury was 9%.

Safety improvements in surgery and other areas can be achieved using reporting systems that capture front-line personnel's reports of complications and adverse events. There is also an emerging interest in the role of patient-reported incidents. In Sweden, patients are entitled to claim economic compensation from the County Councils' Mutual Insurance Company if they believe that they have sustained an injury as a result of their treatment. This national no-fault insurance system is unique to the Nordic countries (Norway, Denmark, Finland, and Sweden). Approximately 9,000 claims are filed every year in Sweden (with 9 million inhabitants) and about half of the claims result in economic compensation for injuries that medical experts at the insurance company consider to be avoidable. The medical experts chosen for each specialty have considerable clinical experience. Orthopedic surgery has the highest number of claims (one quarter of the total) that are compensated by the insurance company (Puck-Härenstam et al. 2009). Injuries in connection with spine surgery tend to be more severe than other orthopedic procedures, usually resulting in disability (Öhrn et al. 2006).

In Sweden, information, including complications, related to spine surgery is reported to a national quality register, the National Swedish Spine Register (Swespine), which was set up in 1992. This register contains preoperative and perioperative baseline data on complications, as well as several validated outcome measures based on patient responses given preoperatively and after 1, 2, and 5 years (Strömqvist et al. 2001, 2005). More than 75% of all 59 departments that perform spine surgery in Sweden report to the register (Strömqvist et al. 2009).

Under-reporting of adverse events is common in both voluntary and mandatory reporting systems (O'Neil et al. 1993, Barach and Small 2000, Wanzel et al. 2000, Sari et al. 2007). For surgical procedures, safety has traditionally been approached as a matter of individual performance; that is, complications represent failure by the surgeon, thus enhancing the risk of under-reporting (Wachter 2008). However, it is widely acknowledged that it is difficult to determine whether an adverse event is caused by faulty treatment or should be seen as an unavoidable complication inherent in the treatment (Wanzel et al. 2000).

We investigated adverse events in spine surgery in Sweden using data from the patient claims data from the County Councils' Mutual Insurance Company register and from the Swespine national quality register. We compared the registers with regard to the number of complications, the degree of disability, and the clinical outcome. In addition, we investigated Swespine's coverage concerning injured patients and complications and whether there were any differences in outcome between injured patients and patients who were not injured.

## Patients and methods

Data were obtained from 2 national Swedish registries: the County Councils' Mutual Insurance Company and Swespine. Data were also gathered by reviewing the medical records. The study period was from 2003 through 2005. The Regional Research Ethics Committee in Linköping, Sweden, approved the study (registration number M41-07).

Data from 208 patient claims attributed to spine surgery during the study period were obtained from the insurance company. This database contains information on discharges from hospital, medical specialty, diagnostic codes, surgical procedure codes, patient age, and sex. The database also contains information on whether the claim was denied or approved. If approved, the type of injury and the degree and consequences of the disability are recorded. Injury type and consequences are classified by the insurance company. The classification is based on injury criteria defined by the law on Swedish patient injury. We received approval from the board of the insurance company to analyze data pertaining to the 208 patients. Of the 208 patient claims, 26 were excluded because the injury was not related to the surgical procedure (e.g. teeth injuries during anesthesia). Thus, 182 claims were analyzed in the study.

The Swespine database includes basic hospital data on age, sex, date of admission, surgical procedure, and discharge. The surgeon's report on the procedure and any perioperative complications and adverse events identified during the hospital stay are also included. Follow-up assessment is performed after 1, 2, and 5 years, and includes several validated outcome instruments such as global assessment, Oswestry disability index, pain (visual analog scale), and SF-36. Surgical success is defined by a report from the patient that they are pain-free or have experienced a major improvement. Questions about complications, reoperations, and patient satisfaction are included in the follow-up assessment.

We used information from the insurance company for analysis of the degree of disability for subjects with compensated claims (Table 1). Information from the insurance company, Swespine, and the medical records was used for analysis of the type of injury, number of complications, and degree of disability. We classified the complications into 7 categories (Tables 2–3).

**Table 1.** Degree of disability and gender distribution for compensated patient claims from the County Councils' Mutual Insurance Company, from 2003 through 2005 (n = 139)

Degree of disability	Male, n	Female, n	Total, n
Period of sick leave but no permanent disability	16	16	32
1–15%	39	46	85
16–30%	5	5	10
Over 30%	4	8	12
Total	64	75	139

We used a structured protocol for analysis of the 182 patient claims. The review was based on the medical records and the files from the insurance company. The files from the insurance company included the patients' self-reported claims, a judgment from the surgeon responsible, and an assessment from the insurance company's medical expert of whether or not the injury had been avoidable.

The injured patients had been treated for degenerative low back problems including disc herniation, spinal stenosis, and mechanical problems such as spondylolisthesis and segmental pain. We compared the outcome for the 182 patients with the results from the Swespine register for all patients with the same diagnoses for the same time period. Information about the injury, mechanism, and degree of disability from the insurance company was compared with the patient's self-reported clinical outcome in Swespine.

Coverage was defined as the proportion of injured patients included in Swespine preoperatively and follow-up was defined as the proportion of patients who had answered the questionnaires at 1 year. The patients registered in the Swespine database and the County Councils' Mutual Insurance Company's claim register were matched by their personal identification numbers.

### Statistics

Exact 95% confidence intervals (CIs) based on the binomial distribution were calculated for the patients who claimed economic compensation, and large-sample-approximated 95% confidence intervals were calculated for patients in the Swespine database. Microsoft Office Excel 2007 and PASW statistics (SPSS) 18.0 were used for statistical calculations.

## Results

The mean age of the 182 patients (51% female) who claimed economic compensation from the insurance company was 50 (13–89) years for men and 53 (18–83) years for women. Of the 182 claims, 139 (76%) were approved and the patients received compensation, i.e. the injury was considered to have been avoidable by the medical experts at the insurance company. Of the patients who received compensation, 54% were women (Table 1).

Of the 182 patients, 119 were found in the Swespine database; thus, coverage in the Swespine register was 62%. Basic preoperative data were missing for 6 of these patients (5%) and follow-up data were missing for 32 of 119 patients (27%). Of the 119 patients in Swespine, 43% had had a registered complication during the hospital stay and 16% had undergone a reoperation during their hospital stay.

The medical-record review revealed 210 complications in the 182 patients who claimed economic compensation. 16% had undergone one or more surgical procedures prior to the injury for which they claimed compensation. Swespine listed 74 complications for the same 182 patients (Table 2). Dural lesion was the most frequent complication, with 56 cases (51 were detected during the surgical procedure and 5 were detected postoperatively). Of the 56 cases with an identified dural lesion, 40 patients received compensation from the insurance company for an avoidable injury. Of the 56 cases, 38 were reported in Swespine, but only 27 had a dural lesion registered as a complication of the surgery (Table 2). In 24 of the 56 cases in which a dural lesion occurred, the patients underwent one or more additional surgical procedure(s). Dural lesion (alone or in combination with other causes) led to a high degree of disability (Table 3). The review also showed that around 10% of the dural lesions were not detected during the surgical procedure, which led to one or more further surgical procedures in all 5 cases.

**Table 2.** The distribution of complications found in medical records regarding patient claims to the County Councils' Mutual Insurance Company and complications reported to Swespine, from 2003 through 2005. (A patient could have more than 1 complication)

Type of complication	No. of complications found in the review of claims to the County Councils' Mutual Insurance Company (n = 182)	No. of complications registered in the Swespine national quality register (n = 119)
Dural lesion	56	27
Infection	34	1
Implant problem	18	9
Hematoma	16	7
Wrong level	8	0
Nerve tear	31	8
Other	47	22
Total	210	74

**Table 3.** Distribution of complications, distribution of combinations of complications, and the degree of disability in compensated patient claims to the County Councils' Mutual Insurance Company, from 2003 through 2005 (n = 139)

Type of complication or combination of complications	Sick leave period but no permanent disability	Degree of disability			
		1–15%	16–30%	Over 30%	Total
Dural lesion	4	12	1	4	21
+ hematoma	0	1	0	1	2
+ nerve tear	1	7	1	0	9
+ implant problem	0	2	0	0	2
+ infection	4	2	0	0	6
Subtotal					40
Infection	8	12	0	1	21
+ hematoma	0	1	0	0	1
+ other	1	0	0	0	1
Subtotal					23
Nerve tear	0	13	3	0	16
+ implant problem	1	4	1	0	6
Subtotal					22
Implant problem	0	4	1	0	5
+ hematoma	0	1	0	0	1
+ other	1	2	0	0	3
Subtotal					9
Hematoma	0	4	2	3	9
Wrong level	3	5	0	0	8
Other	9	15	1	3	28
Total	32	85	10	12	139

Postoperative infection was the second most common complication; 34 postoperative infections were identified in our review of the medical records. Only 1 of these cases was registered in Swespine.

In the 1-year follow-up assessment of the injured patients registered in Swespine, 77% of patients reported complications, and reoperations were reported by 33% of the patients, but reoperation in this context includes every additional lumbar spine operation irrespective of the relationship to the first operation. Full satisfaction with the operation was reported by 30% of the injured patients (CI: 21–41), as compared to 68% (CI: 67–69) in Swespine.

In general, clinical outcome was worse for the group of patients who claimed economic compensation than for the Swespine patients. Global assessment was worse, with a success rate of 52% (CI: 41–63) for back pain for those who claimed economic compensation as opposed to 63% (CI: 62–64) for the Swespine patients—and 45% (CI: 34–56) vs. 64% (CI: 63–65) for leg pain. The outcome for the study group regarding the other outcome variables was worse than the corresponding results from Swespine (Table 4).

**Table 4.** Average score for clinical outcome and the average score for improvement after 1 year for all patient claims and for the patients with dural lesions, compared with general data from Swespine from 2003 through 2005

Outcome variable	All patient claims including patients with dural lesions (n = 87)		Patient claims for patients with dural lesions (n = 20)	Swespine (n = 7,819)
	Average score (SD)	95% CI	Average score (SD)	Average score
Back pain (VAS), 1 year	40 (29)	34–46	50 (30)	31
Back pain, improvement (VAS)	22 (32)	15–30	8 (30)	23
Leg pain (VAS), 1 year	43 (30)	36–49	45 (29)	29
Leg pain, improvement (VAS)	19 (39)	10–28	19 (31)	30
EQ, 1 year	0.38 (0.34)	0.30–0.46	0.35 (0.29)	0.62
EQ, improvement	0.19 (0.38)	0.10–0.28	0.10 (0.26)	0.33
ODI, 1 year	37 (19)	33–42	41 (18)	26
ODI, improvement	11 (22)	5–17	6 (16)	20

VAS: visual analog scale; EQ: EuroQol; ODI: Oswestry disability index.

## Discussion

We found a considerable degree of under-reporting of surgical complications. One third of the 182 patients who claimed economic compensation from the County Councils' Mutual Insurance Company were not found in the Swespine database, and of the 210 complications associated with these 182 claims, 136 (two-thirds) were not listed in Swespine. Our findings are in accordance with the results from a study by Franneby et al. (2008) of another national Swedish quality register, the Swedish Hernia Register. These authors showed that adverse events after surgery for inguinal hernia were reported to the Swedish Hernia Register to a far higher degree by patients using self-report questionnaires than by healthcare providers. Four-fifths of the 391 patients who reported adverse events by questionnaire were identified in the Swedish Hernia Register. The overall purpose of Swedish quality registers such as the Swedish Hernia Register and Swespine is to increase our knowledge of the frequency, costs, and effects of treatment to facilitate improvements in the quality of healthcare. However, our findings and those of Franneby et al. (2008) show that there is considerable under-reporting of complications to these registries that must be accounted for when interpreting the data.

Patient claims reported to the insurance company were analyzed by experts in orthopedic surgery, which means that a complaint that leads to compensation is probably correctly judged as an avoidable adverse event. It is not known whether the under-reporting of patients with adverse events in the Swespine register is systematic or whether it is simply due to incomplete registration. To investigate this, a control group of patients experiencing satisfactory results after the surgical procedure must be compared with a group of patients reporting complications during the surgical procedure.

Dural lesion was the most common complication, but we found twice as many in the medical records (n = 56) as in the Swespine register (n = 27). In many cases, the injury was documented in the patient's file and reported to the insurance company but was not included in the Swespine report, although other patient data were properly documented. It is likely that sometimes surgeons do not consider a dural lesion to be an important complication. A dural lesion may be found intraoperatively, but not be reported either because the surgeon is not willing to admit a mistake or because the problem is considered to be solved. The dura mater may be damaged accidentally during resection of the ligamentum flavum or during direct decompression and laminectomy. The incidence of dural lesions during spine surgery is estimated to range from 0.3% in index procedures to 17% in revision surgery. 7% of lesions have been reported to go unnoticed at the time of surgery (Stolke et al. 1989, Wang et al. 1998, Le at al. 2001). Nerve root injuries often occur with dural lesions, and the dural tear might precede root damage. Repair of dural defects may also compromise the nerve root at that level (Gupta and Kahn 2006).

A study from 2011 has shown a prevalence of unintended dural lesions of 1.1% (Ahn et al. 2011). Strömqvist et al. (2010) found an incidence of dural lesions of 2.7% in lumbar disc herniation surgery. According to the Swedish Patient Register of the National Board of Health and Welfare, 11,489 spine surgery procedures were performed in Sweden during the study period (around 3,830 annually). With an incidence of 2.7%, dural lesions would occur in approximately 100 patients who undergo spine surgery every year. Our results indicate that this figure is too low because many dural lesions are not reported.

We identified 34 postoperative infections in our review of these cases, but only 1 of them had been registered in Swespine. One explanation for the missing registration of infections in Swespine would be that a postoperative infection often occurs some time after the patient has been discharged. Thus, this information would not be available when the protocol is filled in directly after the discharge of the patient. The first follow-up in Swespine takes place 1 year postoperatively, at which time the infection may have been treated and the infection episode forgotten. However, changes have been made to the questionnaire in Swespine since we undertook this study. Direct questions concerning wound infections are now included, which should reduce the amount of under-reporting. However, identification of adverse events would probably be better if the first follow-up took place after 4–6 weeks instead of 1 year after the operation. This follow-up could be performed by a research nurse using a structured interview guide.

We found that the injured patients had worse clinical outcome than all patients registered in Swespine. In particular, improvement regarding leg pain and EuroQol score were impaired after dural lesions associated with a high grade of disability. This finding contrasts with a recent study by Strömqvist et al. (2010) that scrutinized Swespine for patients with dural lesions reported in connection with lumbar disc surgery. These authors did not find inferior clinical outcome at follow-up, and their results were better than the results seen in our patients. We analyzed a subgroup, namely those with a reported injury. The registry group is probably another subgroup: those remembered by the surgeon and considered worth recording. This could be one explanation for our results. Moreover, the poor coverage and follow-up in Swespine probably affects the results.

In conclusion, this study shows that a full understanding of patterns of adverse events and outcomes after spine surgery requires analysis of data from more than one source. Self-reports by patients and patient claims are important sources of information, and can provide us with better knowledge—and a more accurate picture—of adverse events and their outcome. Dural lesions and infections are common injuries in spine surgery, but they are not well recorded in the national Swespine quality register, despite being an important reason for postoperative disability.
